# Tectonic Keratoplasty to Restore the Bulbar Wall after Block Excision of Benign and Malignant Intraocular Tumors

**DOI:** 10.1155/2019/4153064

**Published:** 2019-05-02

**Authors:** Emilio Balestrazzi, Luigi Mosca, Maria Antonietta Blasi, Maria Ilaria Giannico, Angelo Balestrazzi

**Affiliations:** ^1^Studio Oculistico Associato Balestrazzi, Rome, Italy; ^2^Department of Ophthalmology, Catholic University, Policlinico Gemelli Hospital, Rome, Italy; ^3^Department of Ophthalmology, Catholic University, Ocular Oncology Unit, Policlinico Gemelli Hospital, Rome, Italy; ^4^Department of Ophthalmology, “Misericordia” Hospital, Grosseto, Italy

## Abstract

**Purpose:**

To report the surgical treatment and follow-up of tumors of the anterior uvea and epithelial cysts of the anterior chamber in 4 patients submitted to block excision and tectonic corneal graft between 2008 and 2012.

**Methods:**

This is a retrospective, nonrandomized case series. Two patients were affected by anterior uveal malignant melanoma, and 2 patients were referred to us for large epithelial iris cysts with anterior chamber angle involvement and partial corneal failure. A simultaneous block removal of the lesion and adjacent iris, cornea (when necessary), ciliary body, and sclera was performed; the resulting defect was covered by a tectonic whole thickness corneal graft. Follow-up ranged from 2 to 7 years (mean time: 5 ± 1.6 MD).

**Results:**

Local control of malignant melanoma was observed during the follow-up, but cataract surgery was planned in both patients and pars plana vitrectomy for vitreous hemorrhage occurred in one case. No recurrence of cysts was detected. After iris cysts excision, a planned second-time surgery was necessary in one patient: optical penetrating keratoplasty, centered on the visual axis, implantation of one refractive IOL (intraocular lens) in the bag, and one cosmetic IOL in the sulcus, to restore the iris diaphragm.

**Conclusions:**

Block excision followed by the tectonic corneal graft seems to be the treatment of choice for selected cases of epithelial cysts of the anterior chamber and anterior uvea melanomas with epibulbar extension. Further surgery, as a second step, could be required to improve functional results of this challenging technique.

## 1. Introduction

Treatment of uveal melanoma includes brachytherapy, proton beam irradiation, TTT (transpupillary thermotherapy), enucleation, and combined therapies. Surgical local resection can be an alternative in selected cases but requires a scleral flap to cover the sclero-uveo-tumoral defect. This technique is, unfortunately, impossible to perform in cases of scleral neoplastic infiltration or extraocular extension. In these cases, the unique possibility of surgical excision is a removal of a sclero-uveo-tumoral block, what makes necessary to cover the whole thickness defect with a new wall. Several tissues have been proposed for this aim: mainly bovine pericardium, human preserved eye bank sclera, and human eye bank cornea, rejected for optical keratoplasty [1–5].

Congenital iris cysts could arise from the pigment epithelium [6] and stroma [7] of the iris.

Acquired epithelial-ingrowth cysts could originate from implanted surface conjunctival epithelium after anterior segment surgery or perforating ocular injury [8]. Several techniques have been performed for the treatment of these potentially blinding lesions: puncture and aspiration [9], diathermy electrolysis [10], irradiation [11], transcorneal cryotherapy [12], argon laser photocoagulation [13], and surgical excision [14–17]. Any treatment should allow a complete eradication of the secretory epithelium to prevent recurrence and enlargement of the cysts and to avoid diffuse epithelisation of the anterior chamber that could induce refractory secondary glaucoma and blindness.

Ciliary and anterior choroidal melanomas and epithelial iris cysts nearly always involve the chamber angle structures, the posterior corneal surface, the anterior iris surface, and the ciliary body. Therefore, a more radical surgical approach by removing the lesion together with all the adjacent full-thickness cornea, iris, ciliary body, and sclera in one block could be indicated to save the eye, remove the pathological tissue, and possibly preserve function.

We describe our surgical technique and the results of 4 patients who underwent block excision with the tectonic corneal graft for anterior uveal melanoma and epithelial congenital iris cysts, with a long-term follow-up.

## 2. Methods

Four patients (3 women, average 35.3 years, and one man, 70-year-old) were referred to the Department of Ophthalmology of the A. Gemelli Policlinic, Rome, Italy, between July 2008 and July 2012 for anterior uveal melanoma (2 eyes) and epithelial iris cysts involving the anterior chamber angle (2 eyes). Patients were sent to the Cornea and Refractive Surgery Service and to the Ocular Oncology Service of the same Policlinic during the entire follow-up (ranging from 2 to 7 years). Digitalized anterior segment photos were acquired preoperatively and postsurgery, with slit lamp examination (CSO, Compagnia Strumenti Oftalmici, Florence, Italy); A/B-scan ultrasound examination and ultrabiomicroscopy (UBM) (Aviso STM, Quantel Medical, Cournon d'Auvergne, France; A/B-scan HiScan Echograph and UBM, Optikon 2000, Rome, Italy) were performed to define the site and the extension of the lesions in each eye. Surgery was performed by the same surgeon (EB). Informed consent was signed by all patients, and the present study and treatment performed adhered to the principles outlined in the Declaration of Helsinki.

### 2.1. Patient 1

**Figure 1:**
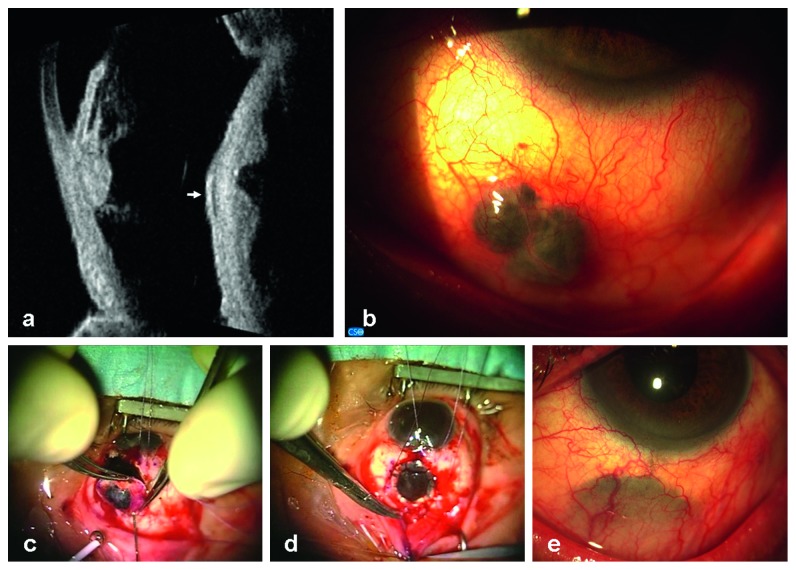
Patient 1 UBM before surgery and after uveo-scleral excision en bloc. (a) Left: longitudinal scan at the 6 o'clock position (L6) shows a solid thickening of the ciliary body and the anterior choroid; the corresponding anterior chamber angle is infiltrated, the acoustic structure is heterogeneous, and the internal reflectivity is medium. Right: transversal scan at the 5:30 o'clock position (T5:30) shows the double dome-shaped morphology of the lesion at the level of the anterior choroid and the circumscribed growth into the overlying sclera, which appears hypoechoic (white arrow). (b) Patient 1: slit lamp examination presurgery. Image shows a confined pigmented nodular growth of uveal melanoma through the sclera, at about 6 mm from the limbus, under the conjunctival layer. (c) After Hanna trephination, block excision is completed with scissors until removing the entire lesion and the overlying infiltrated sclera. (d) Tectonic corneal graft covers the scleral defect and is sutured with 16 interrupted Nylon 10.0 sutures. (e) Corneal graft is well integrated into the sclera, under the conjunctiva, about 4 years after surgery.

Patient 1 was a 70-year-old man. He presented to our clinic in September 2008. Ultrasound examination and UBM showed in the left eye a solid irregular-shaped thickening starting at the level of the choroidal layer of the preequatorial part of the eye, between the 5 and 6:30 o'clock positions (Figure 1(a) left), associated with circumscribed extraocular growth, into the overlying sclera under the conjunctiva, 2 mm thick (Figure 1(a) right). This nodule was at about 6 mm from the limbus (Figure 1(b)); the adjacent ciliary body was thickened, and the basal diameter of the entire lesion was large about 13 mm. The corresponding anterior chamber angle was infiltrated, the acoustic structure was heterogeneous, and the internal reflectivity was medium; transversal diameter was 8.09 mm, longitudinal diameter was 8.89 mm, and the thickness was 3.5 mm. The diagnosis of melanoma involving the ciliary body and the anterior choroid extended into the sclera was made.

The patient was submitted to scleral full-thickness block excision (8.50 mm large) of tumor, combined to the corneal graft, 16 interrupted Nylon 10.0 sutures (Figures 1(c)–1(e)). Histological analysis confirmed the diagnosis of pigmented epithelyoid cell melanoma.

### 2.2. Patient 2

**Figure 2:**
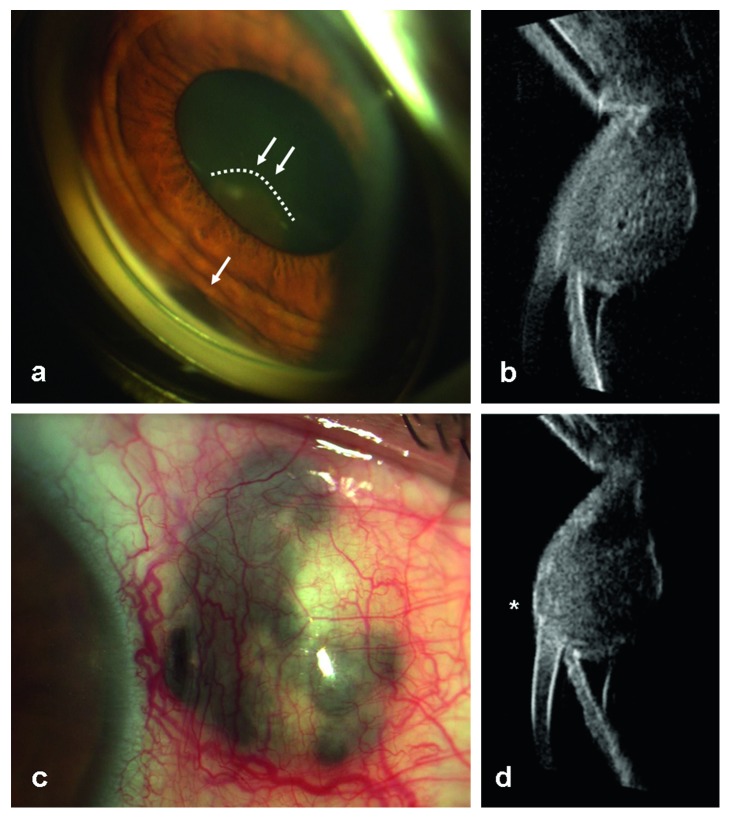
Patient 2 before treatment. (a) Gonioscopy shows a pigmented fusiform thickening of the iris root (white arrow) and a dome-shaped pigmented mass behind the pupil foramen (dashed line and double white arrow). (b) UBM longitudinal scan at the 2:30 o'clock position (L2:30) showing a circumscribed dome-shaped mass at the level of the ciliary body, extended to the iris root, which touches the anterior surface of the lens. (c) Scleral thinning and prolapse of the uveal tissue at the limbus, one year after brachytherapy. (d) UBM L2:30 scan shows a local scleral extension of the tumor, under the conjunctival layer (white asterisk).

She was a 48-year-old woman affected by a pigmented fusiform thickening of the iris root in the left eye, as shown by gonioscopy (Figure 2(a)). UBM revealed the presence of a well-circumscribed dome-shaped mass at the level of the ciliary body, between the 2:30 and 3 o'clock positions (Figure 2(b)). The acoustic structure was almost homogeneous, the internal reflectivity was medium, and the lesion was 8.18 mm large, 7.74 mm long, and 4.88 mm thick. It was classified as melanoma of the ciliary body, involving the adjacent iris root.

In July 2008, she underwent brachytherapy (^106^Ru): 100 Gy at the apex, dose rate: 79.09 cGy/hr; total rate at the sclera: 531.1 Gy, dose rate: 420.1 cGy/hr.

One year later, the patient showed a scleral thinning and a prolapse of uveal tissue at the limbus, corresponding to the site of the preexisting lesion (Figure 2(c)). UBM showed a local scleral extension of the tumor, under the conjunctival layer, 0.88 mm thick (Figure 2(d)).

**Figure 3:**
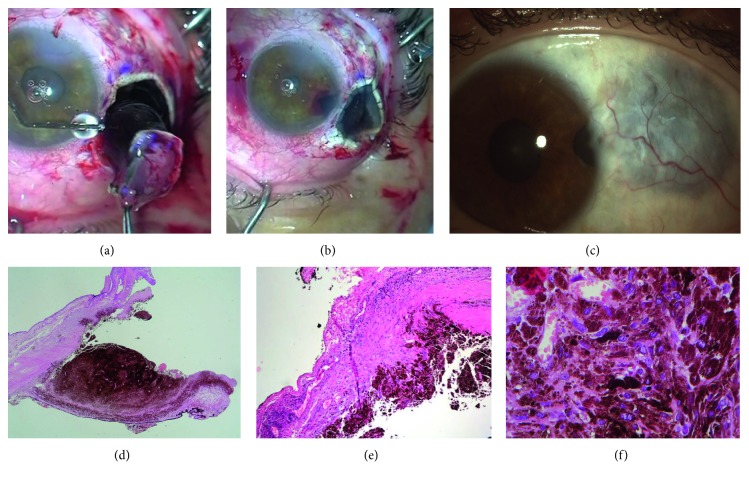
Patient 2 block excision of sclera and ciliary melanoma. (a) Removal of the ciliary melanoma en bloc after marking the margins and after Hanna trephination. (b) Corneal graft is sutured to the adjacent sclera; basal iridectomy. (c) Corneal graft is in situ 7 years after surgery well visible under the conjunctiva. (d) The perfect clearance of trephination on healthy scleral and ciliary body margins. (e, f) Postirradiation necrotic aspect of spindle melanoma's cells.

BCVA was 20/20. The patient underwent a radical block excision of sclera, ciliary melanoma, and adjacent iris tissue, by a peripheral iridectomy performed through the opening of the sclera, combined to the corneal tectonic graft (8.50 mm large), 16 single stitches (Figures 3(a)–3(c)).

Histological analysis confirmed the diagnosis of spindle cell melanoma (Figures 3(d)–3(f)).

### 2.3. Patient 3

**Figure 4:**
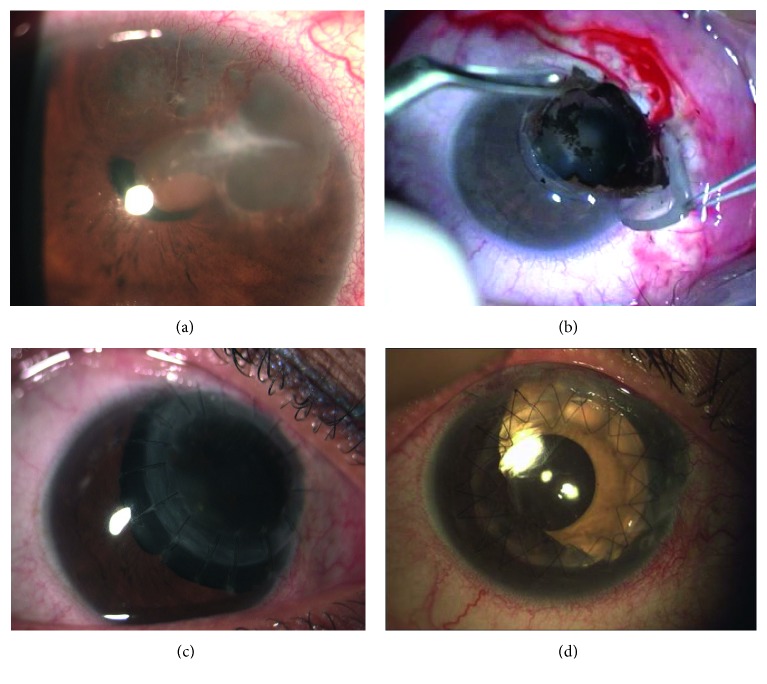
Patient 3 before and after surgery. (a) Large epithelial cysts of the iris involving the upper nasal quadrant of the right eye. Cysts stretch the pupil foramen and cause circumscribed corneal failure. (b) Block excision of the cysts, iris, cornea, and limbal sclera after Hanna trephination. (c) Eccentric corneal graft is clear one-year postoperatively, and sutures are still in situ; a large iris coloboma is observable. (d) Two months after penetrating keratoplasty and cataract surgery. Photo shows that the graft is clear and the cosmetic IOL is well positioned in the sulcus; double continuous Nylon 10.0 suture is in situ.

Patient 3 was a 42-year-old woman affected by epithelial cysts of the iris in the right eye, which had increased in size in the last few years, affecting the three upper-nasal clock hours of the anterior chamber, with related partial corneal failure (Figure 4(a)).

A block excision of the cysts, iris, cornea, and limbal sclera, including the angle, was performed in May 2010. A corneal graft (8.50 mm large), 16 interrupted Nylon 10.0 sutures, was contemporary sutured to the sclera and residual cornea to restore the bulbar wall (Figures 4(b) and 4(c)). About 3 years later, she underwent a penetrating keratoplasty centered on the visual axis, combined to phacoemulsification of the cataract and refractive IOL implantation in the capsular bag, together with cosmetic neutral IOL implantation in the sulcus (Figure 4(d)).

### 2.4. Patient 4

**Figure 5:**
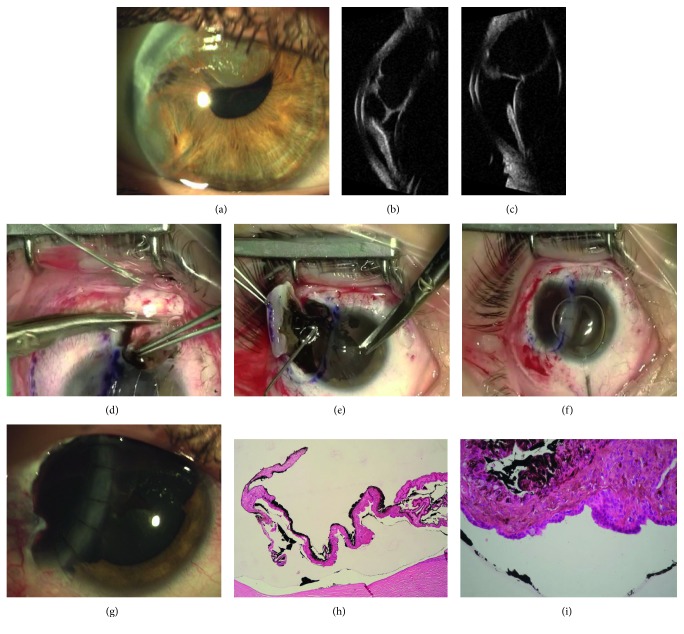
Patient 4 before surgery and after uveo-sclero-corneal block excision. (a) Epithelial iris cysts between the 8 and 12 o'clock positions of the right eye, involving the anterior chamber angle, partially stretching the pupil foramen. (b) UBM transversal scan at the 11 o'clock position (T11) showing multiple optically empty cysts into the anterior chamber occluding the chamber angle, in contact with the corneal endothelium. (c) UBM axial scan at the 12 o'clock position (A12) showing how the large cyst touches the equator and the anterior surface of the lens. (d) Removal of the major cyst at 12 o'clock after creating a scleral fornix-based flap and a limbal incision to enter into the anterior chamber. (e) Block excision of cysts from 8 to 11 o'clock after core dry vitrectomy and manual incision of the marked sclera with a diamond knife. Viscoelastic substance is injected into the anterior chamber. (f) Corneal graft is sutured to the sclera and to the patient's cornea with interrupted Nylon 10.0 sutures. (g) Corneal graft is clear two years after surgery. Sutures are still in situ. (h, i) Perfect integrity of the cyst's wall after block excision, outlined by the inner surface of cornea, angle, iris, and ciliary body. The empty, clear spaces outlined by the cyst's walls are related to the cyst's serous content.

She was a 17-year-old girl who was sent to our clinic for diffuse recurrence of epithelial iris cysts in the right eye, which involved the anterior chamber structures of the temporal side between the 8 and 12 o'clock positions (Figure 5(a)). She had previous surgical puncture and aspiration of the cysts when she was 7 years old. UMB showed multiple iris cysts extended into the anterior chamber in contact with the corneal endothelium; major cysts touched the equator and the anterior surface of the lens, occluding the anterior chamber angle and stretching the pupil foramen (Figures 5(b) and 5(c)). In July 2012, the patient underwent surgery. Removal of cysts at 12 o'clock was possible by creating a scleral rectangular fornix-based flap, 5 × 4 mm large, and entering into the anterior chamber through a limbal linear incision, as in transcleral local resection (Figure 5(d)). Corneal tissue over these cysts, which was not involved, was preserved from excision and an anterior synechiolysis preceded uveo-scleral cysts block removal. Scleral flap was then sutured with five interrupted radial Nylon 10.0 stitches. An uveo-sclero-corneal block excision, including cysts from 8 to 11 o'clock, was then completed, after a core, decompressive, dry pars plana vitrectomy, and covered by the tectonic corneal graft, 8.50 mm large, 16 single stitches (Figures 5(e)–5(g)). Histopathology confirmed the epithelial origin of the cysts (Figures 5(h) and 5(i)).

### 2.5. Surgical Technique

The following are the main points of our surgical procedure. Surgery was performed under general anesthesia combined with controlled arterial hypotension. After removing the overlying conjunctiva and performing a transillumination by the optic fiber, a dermographic pen was used to mark the corneoscleral tissue to be excised. Transcleral monopolar diathermy was placed on the sclera around the tumor-free margins before excision, for all cases, to obtain haemostasis. Decompression of the vitreous chamber was made prior to block excision in all cases, with a pars plana core, dry vitrectomy to prevent expulsive hemorrhage, allowing bulbar hypotonia. The area over the lesion was trephined through the cornea and/or the sclera using the Hanna or Franceschetti trephine, if possible, or manually, with a diamond knife or 15° knife, when lesions were too large or the edges quite irregular, and completed with scissors. During the procedure, the injection of viscoelastic substances into the anterior or posterior chamber, as necessary, enabled to restore the bulbar volume. A blunt spatula was used for anterior synechiolysis. The resulting defect of the globe was covered by a corneal graft and sutured with interrupted Nylon 10.0 stitches. After surgery, the eyes were medicated by topical antibiotics, corticosteroids, and cycloplegic eye drops, during about a month.

## 3. Results

**Figure 6:**
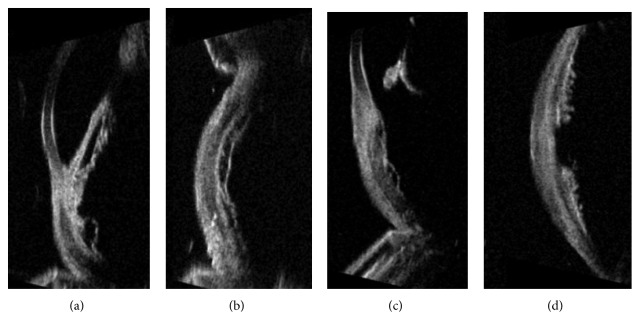
UBM scans after surgery. (a) Patient 1, one-year postsurgery: longitudinal scan at 5:30 o'clock position (L5:30) shows the persistence of circumscribed solid thickening of the ciliary body and the iris root along the edges of the surgical coloboma. (b) Patient 1, one-year postsurgery: transversal scan at 5:30 o'clock position (T5:30) how the corneal graft is well included into the adjacent sclera. Corneal tissue presents with an internal lower reflectivity. (c) Patient 2, three years after surgery: longitudinal scan at 2:30 o'clock position (L2:30) shows the coloboma of the iris and the anterior part of the tectonic corneal patch. (d) Patient 2, three years after surgery: transversal scan at 2:30 o'clock position (T2:30) shows the coloboma at the level of the ciliary body with no local recurrence of tumor.

3 months after surgery, patient 1 developed vitreous hemorrhage and cataract, so he underwent pars plana vitrectomy and phacoemulsification with IOL (intraocular lens) implantation in the capsular bag. 3 months later, adjuvant brachytherapy (^106^Ru) was performed. UBM showed the persistence of solid thickening of the ciliary body and the iris root along the edges of the surgical coloboma (Figure 6(a)) but remained unchanged until the last follow-up visit, in February 2013, more than 4 years after surgery. Hepatic ultrasonography was negative. Tectonic cornea appeared well integrated to the adjacent scleral tissue (Figure 6(b)). Subsequently, two cycles of SLT (selective laser trabeculoplasty) were necessary for ocular hypertension, 3 and 13 months later, respectively, even if topical hypotonic therapy was also prescribed to normalize intraocular pressure. Best corrected visual acuity (BCVA) decreased from 20/66 at 2 years from surgery, to perception of hand movement at the last follow-up visit for corneal failure.

In case 2, postoperative UBM controls showed no signs of local recurrence of the tumor (Figures 6(c) and 6(d)); corneal patch appeared well in situ, until the last follow-up, 7 years later. BCVA was 20/20 for many years with a cylinder of −1.50 [18]; BCVA got worse during the last visits (20/25) because of the onset of posterior cortical cataract. After cataract surgery, BCVA was 20/20, almost 6 years after block excision. Hepatic ultrasonography was negative.

Patient 3 showed a BCVA of 20/100, 6 months postoperatively, but irregular astigmatism, due to the leukoma of the previous tectonic keratoplasty on the visual axis, iris coloboma, cataract, and photophobia affected quality of vision. About 3 years later, she underwent a penetrating keratoplasty centered on the visual axis, combined to phacoemulsification of the cataract and refractive IOL implantation in the capsular bag, associated with cosmetic neutral IOL implantation in the sulcus (Figure 4(d)). Corneal graft remained clear until the last follow-up visit in March 2015, 5 years after block excision; BCVA was 20/28, and no recurrence of the cysts was detected at UBM examination, with no photophobia.

Unlike the previous case, patient 4 BCVA was 20/25 one year after surgery and 20/22 two years postoperatively. The iris coloboma was covered by the peripheric leukomatous margin of the tectonic keratoplasty and did not affect the quality of vision. For this reason, no second-time surgery was necessary until the last follow-up in July 2015 because the patient was largely satisfied. No recurrence of cysts was detected.

## 4. Discussion

The purpose of every treatment of uveal malignant tumors is the complete eradication of the pathological tissue, the prolongation of life, and the preservation of globe and vision, if possible [8]. Iridectomy has been proposed for small-to-medium-sized iris tumors not involving the anterior chamber angle [19, 20], while choroidal and posterior ciliary body tumors are often treated with brachytherapy [18, 21]. Enucleation represents the treatment of choice for large uveal tumors not susceptible to local resection, brachytherapy, or proton beam irradiation [22, 23]. How to manage uveal tumors involving the anterior chamber angle, which are a selected minority of cases, is still now controversial. Radiation therapy may lead to several complications, such as cataract and irreversible secondary angle closure or neovascular glaucoma and rubeosis iridis due to ischemia of the anterior segment [24, 25].

Local excision of tumors of the ciliary body was already achieved in 1911 by Zirm with the description of iridocyclectomy [2] and by Schubert, in 1925, who introduced choroidectomy [26]. Later reports was followed in [3, 19, 27, 28] and others, such as the description of partial lamellar sclerouvectomy by Foulds and collegues [4] and Shields and coworkers [5]. These surgical procedures allow a partial block excision because they preserve the superficial lamellar scleral flap. As suggested by Donders [29], 90% of malignant uveal melanomas invade the sclera histopathologically, with possibility of local tumor recurrence. For this reason, Damato proposed adjuvant brachytherapy in all cases of transcleral local resection. Therefore, the technique of radical block excision en bloc of tumor together with all layers of the adjacent cornea and sclera has developed over time, in cases of macroscopic scleral invasion. According to Naumann et al., this procedure is indicated when tumor involves the pars plicata of the ciliary body up to 4-5 h (120°–150°); the globe tolerates block excision of up to 18 mm diameter, and collateral retinal detachment is not a contraindication for surgery [30].

In our cases, we perform block excision for local scleral and epibulbar tumor extension. The diameter was 8.50 mm in both cases, even if patient 1 presented with a larger size lesion which involved the ciliary body and the anterior choroid, up to an average width of 13 mm. We tried to preserve the globe from enucleation, with the patient's consent, who refused enucleation, but we felt adjuvant brachytherapy necessary to rescue adjacent tissues; thus, it was performed 3 months after surgery. Unfortunately, visual function was not maintained because of secondary refractory glaucoma which led to corneal failure, but tumor growth was locally controlled.

The histopathogenesis of congenital nonpigmented epithelial iris cysts is still not clear; probable mechanisms include the developmental entrapment of the surface ectoderm, the neuroectoderm, or the mesoderm during the formation of the lens vesicle [31–34]. An additional hypothesis has been suggested [35]: a perforating limbal scar due to an occult perforation of the globe with the amniocentesis needle, as detected by histological examination, could support the idea that occult trauma occurring prenatally, or at birth, or during early infancy, might induce the development of iris cysts.

Histopathology confirmed the epithelial origin of the cysts in our two patients (Figures 5(h) and 5(i)).

Acquired epithelial-ingrowth cysts after perforating trauma, or anterior segment surgery, seem to arise from implanted corneal, limbal, or conjunctival surface epithelium, even if it is not still clear why cystic ingrowth develop in some cases and diffuse sheet-like epithelial ingrowth in other ones [8, 36, 37].

While cysts of the iris pigment epithelium are often stable, or grow very slowly over several decades, stromal cysts, which nearly always cover the posterior corneal surface and the anterior surface of the ciliary body, tend to enlarge over time. These observations indicate that a complete eradication of iris cysts can be achieved only by simultaneous en bloc removal of the lesion and the adjacent iris, the pars plicata of the ciliary body, and the cornea and sclera that serves as a shell for the proliferating epithelium [38]. Indications for treatment of both congenital and acquired iris cysts include documented growth, pain, rupture of the cyst, iridocyclitis, secondary glaucoma, corneal clouding, and worsening of vision [39, 40].

Using this full-thickness corneoscleral-iris resection, including the whole cysts, we could treat our two cases successfully, without manipulating the delicate cyst wall, and the epithelium was completely excised. In fact, eventual epithelial residues can continue to secrete, producing new enlarging cysts. Although this radical procedure could induce postoperative complications [38, 41], there are no contraindications to a second-time surgery.

When the eccentric tectonic corneal patching causes irregular high astigmatism and/or with a flange located on the visual axis, we suggest to perform, in a second time, a centered penetrating keratoplasty, to eliminate the leukomatous scar in the visual axis, to partly correct the refractive defect, and to improve the visual acuity. In case of large iris coloboma associated with photophobia or visual impairment in photopic conditions, a cosmetic iris diaphragm or a cosmetic refractive or neutral IOL could be useful combined with cataract surgery, if necessary. After cataract extraction, we use to implant IOL in the capsular bag, firstly, but when the stability of the zonule is not sure, we prefer to implant IOL in the sulcus or through scleral fixation.

The timing of following second-time surgery must be decided carefully, according to necessity. We preferred to complete surgery not before three years after the first treatment in patient 3.

The choice of tissue to be used for this en block surgery is still not uniformly defined. Scleral human graft is a readily available eye bank, easily preserved for months, strong, flexible, and easy to handle; its natural curvature allows it to blend with host sclera. It is avascular and well tolerated with the scarce inflammatory reaction. Nevertheless, failure of scleral homografts has been reported because of the lack of vascularization and biological integration, with subsequent necrosis and melting [42]. Furthermore, we are still not aware of any disease transmission from sclera because segments of human immunodeficiency virus genome have been identified by the polymerase chain reaction in preserved human sclera [43]. Treatment of bovine pericardium with sodium hydroxide eliminates the prions by hydrolysis and viruses by destroying nucleic acids and makes the collagen easily resorbable facilitating biointegration [43]. Nevertheless, chemosis and extrusion are described postoperative complications following the use of bovine pericardium for the wrapping of hydroxyapatite implants and Ahmed devices. The underlying mechanism responsible for wound dehiscence is unclear, possibly caused by an inflammatory response [44].

Conversely, tectonic corneal graft failure and melting is really improbable after block excision because corneal tissue becomes early vascularized and biologically integrated and fits perfectly to the adjacent sclera. As suggested by Balestrazzi and Lafronza in 1973, cornea loses the privilege of avascularity because of a triggering event (metabolic disorders and factors deficiency, inflammatory, traumatic or degenerative processes, phthisis, glaucoma, long-standing retinal detachment, and corneal transplantation). Subsequently, corneal stroma becomes less compact, and it is easily reached by the surrounding preexisting and new vessels that penetrate inside. The lack of oxygen induces an anaerobic metabolism which probably causes the accumulation of vasoactive metabolites or the release of vasoactive substances from mast cells that abound in the sclera and conjunctiva. Following oedema and vasoactive action of these factors could stimulate angiogenesis and the spread of the new vessels into the graft [45]. This mechanism could favour the survival of the corneal graft over time and its intrinsic structural strength which makes cornea a valid and effective tissue to restore the bulbar wall.

According to Heidel et al. [46–48], this procedure could provide tissues not only for histopathologic diagnosis but also for new diagnostic procedures such as molecular profiling with next-generation sequencing for the detection of hotspot mutations in melanoma-related genes. These procedures could help to identify and select high-risk patients for immuno-oncology therapies and trials [46].

In conclusion, this small series of block excision does not allow any statistically significant comparisons with other therapies because there is no control group.

Due to the low incidence of severe intra- or postoperative complications of this technique [38, 41] and the favourable survival of neoplastic patients [19, 20, 28, 49], even if anatomical and visual results appear to be variable [41], block excision with the tectonic corneal graft may be the treatment of choice for a selected minority of anterior uveal tumors involving the sclera and the angle, or with extraocular extension, and for cystic and epithelial ingrowth of the anterior chamber.

Surgical training is required to perform this kind of surgery; further studies and a larger number of patients will be necessary to better define the inclusion criteria, the surgical approach, and longer follow-up results.

## Data Availability

The main part of the data used to support the findings of this study are included within the article. Other data used to support the findings of this study are available from the corresponding author upon request.
